# A Study of Anatomy Teachers' Perception and Acceptance of the Anatomage Table Technology and Digital Teaching Materials in the Training of Medical and Allied Health Students

**DOI:** 10.7759/cureus.32163

**Published:** 2022-12-03

**Authors:** Joshua Ola Owolabi, Robert Ojiambo, Daniel Seifu, Arlene Nishimwe, Ornella Masimbi, Emmanuel Okorie, Darlene Ineza

**Affiliations:** 1 Anatomy and Neuroscience, University of Global Health Equity, Kigali, RWA; 2 Physiology, University of Global Health Equity, Kigali, RWA; 3 Biochemistry, University of Global Health Equity, Kigali, RWA; 4 Basic Medical Sciences, University of Global Health Equity, Kigali, RWA; 5 Simulation and Skills Center, University of Global Health Equity, Kigali, RWA; 6 Medicine, Doctors Without Borders, Brussels, BEL

**Keywords:** innovations, anatomical education, edtech, anatomage, africa

## Abstract

Background: The Anatomage Table is a modern technology that is used to enhance the teaching of human anatomy and related basic medical sciences to medical and allied health students. Its use is gaining popularity. This study considered anatomy teachers' perception and acceptance of the Anatomage Table technology and digital teaching materials in the training of medical and allied health students in African countries.

Materials and methods: Validated questionnaires were used. Altogether, 79 respondents fully participated in the study, with all African regions being represented as follows: Ghana, Nigeria (West Africa), Ethiopia Kenya Rwanda (East Africa), Namibia, South Africa, Zambia (Southern Africa), Egypt (North Africa), and Sudan (Central Africa). Responses were obtained from the electronic Google form, organized on Excel spreadsheets, and analyzed using the SPSS statistical software version 23.0 (IBM Corp, Armonk, NY).

Results: In terms of proportion, 29.1% of respondents reported that they had some level of mastery in using the Anatomage Table; with 6.3% of all the participants reportedly having a high mastery of this technology, 12% and 6% reported that they had an average mastery and low mastery levels, respectively. Participants' rating of their level of agreement with whether the Anatomage Table is a useful EdTech showed that 54.4% of them strongly agreed while 27.8% just agreed. The majority considered the use of the Anatomage as a means of embracing the global culture of technology-in-medical sciences (87.3%).

Conclusion: Most respondents would accept the technology as a complementary tool to support the existing traditional practices, especially cadaveric.

## Introduction

The Anatomage Table (AT) is a modern educational technology (EdTech) that is used to enhance the teaching of anatomy to medical and allied health students. It has a number of advantages including the use of life-like images that were obtained from human bodies using high-quality and high-resolution imaging techniques. Being a software-enabled technology, it could be easily manipulated to serve as a teaching tool with atlas-like images of the human body as well as a digital dissections tool. Furthermore, it might also provide high-quality information during anatomy lessons. In addition to human anatomy images, it also has a number of vertebrate images that could be useful for veterinary medicine as well as comparative anatomy and physical anthropology.

Anatomage as an innovation and medical education technology can be used to enhance medical education in specific ways and contexts. In the context of basic medical sciences education, it has been used as a teaching aid facility to teach anatomy to medical and allied health students, a digital dissection facility, an assessment facility, a case review facility, an anatomical prosection and demonstration, and in a few other special instances including simulation of body functions and phenomena, depending on the setting and user’s skills and experience. For instance, when the Anatomage is used as a dissection facility, it is expected to digitally achieve what could be achieved with traditional dissection procedures with possibly more dexterity and flexibility. It can be used to replace or complement traditional dissection activities, especially when there is a shortage of human cadavers for dissection or a lack of adequate facilities and logistics to meet the requirements in terms of the number of sessions and dissection hours. It can also be used as a teaching tool all by itself in the forms of functional and clinical anatomical simulations, demonstrations and illustrations, or digital atlases and resources. 

This study aims to investigate the perception of anatomy teachers in African schools about the AT technology as well as their openness to technology, otherwise termed acceptance of technology as a medical education tool. Specific objectives included the need to determine the perception of the Anatomage technology by African anatomy teachers in medical and allied health institutions and the factors influencing such perceptions, the level of acceptance, potential benefits of the technology to educators and learners, and existing limitations to the optimal use of this technology.

The AT provides a digital three-dimensional (3D) full-size image of the human male and female bodies which have been re-constructed from scans of fresh and frozen human cadavers and programmed in manners that allow virtual scalpel dissection as well as the visualization of various body structures, organs, and systems in 3D patterns, giving the best possible presentation of the human body. It is very effective for teaching students with close-to-reality perception and for stimulating students in the learning process [1]. Anatomical dissections have always been considered an essential aspect of anatomy teaching to medical and allied health sciences students [2]. Recent developments have introduced the use of interactive software that is enabled on devices including customized, desktop and laptop computers as well as certain hand-held devices. Examples of computer-enabled software include Complete Anatomy and Zygote. Each appears to have its own variety of unique features and strengths, and they somewhat serve as digital, dynamic, and more interactive versions of the traditional atlases.

The Anatomage teaching technology is a computerized table-like facility that was developed by a 3D medical technology company located in San José (California) in conjunction with Stanford University’s Clinical Anatomy Division. The AT works as a fully interactive multitouch screen, which enables the virtual interactive dissection of the full-size human body, moving through layers of tissue or using a virtual knife to cut away and see the structures inside [1,3,4]. AT or any other similar EdTech is yet to be popularly used in most African countries and the rest of the developing world. A major factor has been postulated to be the cost of such technology [1]. This might not be enough justification considering its potential benefits [5]. However, other potential factors that might not yet be investigated might include the level of awareness about the technology and similar ones, the challenges with introducing and integrating new technologies into somewhat traditional and stereotypical curricula and pedagogies, as well as the technical know-how that might be associated with the tools. While these might be reasonable challenges, it is very important to note that medical education is rapidly advancing, and medical and health professionals who are expected to meet the world standard of quality and needs should be trained using world-class, advanced, and technology-inclined methods not only to give them the best learning opportunities and environment but to also indoctrinate them into the global culture of technology which has become indispensable to cutting-edge research and services provision. This study is a purposeful attempt to investigate acceptance, perceptions, and the associated factors in the African context toward advancing medical education in Africa and globally.

## Materials and methods

Every aspect of the development to the implementation of this research was completed virtually. A mixed methodological approach was applied to maximally explore our research questions. It was based on a sequential explanatory design method. First, a total population study involving a quantitative online survey using a structured questionnaire was followed by a focused group discussion for consenting survey participants to further understand institutional contexts and the teaching teams’ perceptions and experiences. Subsequently, the head of departments or Anatomage unit coordinators were also engaged in an in-depth interview where possible. This report considers only the quantitative data.

The quest to have an African narrative of African anatomy teachers’ views, perceptions, and experiences guided our study population's engagement with both user/non-user populations and new user populations. In our mapping, we divided the continent first into its regions from where we selected a representative country (ies) and institution (s) - West Africa, East Africa, Southern Africa, Northern Africa, and Central Africa. Though it was a non-random engagement of anatomy teachers’ clusters in each school, certain criteria were employed to ensure the matching of study population characteristics, viz:

1. A medical school that uses Anatomage was matched with a non-user school in each country or region.

2. Consideration of an institution that is in the process of installing the AT; applicable to an institution in Ghana.

3. At least a medical school was mapped in each of the five regions of Africa.

4. First, targeted representative countries in each region, and thereafter representative institutions were selected.

The study prioritized a university that is currently using Anatomage to teach anatomy, then identified a contemporary institution that does not have Anatomage as its match or control. In each situation, all members of the anatomy teaching team were contacted and given the opportunity to give informed consent for participation in the study. The study populations were reached, engaged, surveyed, and ultimately interviewed in phases. In each of these phases, the target population was reached through a modified snowball sampling or chain referral sampling method in which we maximized the diverse backgrounds and connections of the study team at the University of Global Health Equity (UGHE) to network reach heads of departments of anatomy or any contacts in the departments of the anatomy of targeted institutions. This was effective because our criteria were clear and the population in question has a network in which lecturers often meet in various fora or associations or on visiting lecturing or examining duties in secondary institutions. We also searched and contacted similar contacts on LinkedIn and through Google search on institutional websites. Email messages, LinkedIn messages, phone texts before phone calls, WhatsApp messages when permitted, Signal and Skype messages, or calls before Zoom meetings were utilized according to the specified or perceived preferences of each contact. Next, we arranged for an informative zoom meeting with the contact persons (s) mostly involving the heads of departments where we shared a PowerPoint synopsis of the project proposal and gave them the opportunity to share their thoughts and consider sharing with their team with all ethical considerations or helping to get contacts of their staff with their consent for us to arrange a similar meeting with all the staff.

We subsequently recruited participants from the representative institutions in the African regions including anatomy and medical educators who met the set criteria in the representative institutions in the 11 African countries. Altogether, 79 respondents fully participated in the study, with all African regions being represented as follows: Ghana, Nigeria (West Africa), Ethiopia, Kenya, Rwanda (East Africa), Namibia, South Africa, Zambia (Southern Africa), Egypt (North Africa), and Sudan (Central Africa).

Administration of questionnaire to the participating faculty members

A structured questionnaire was developed into a Google form and administered to the respondents who meet the inclusion criteria mainly via email messaging. They were also given adequate time to consider the questionnaire, request any clarification via email exchange, and submit their responses.

Study materials and tools

The study tools we used in our quest to sufficiently answer the research objectives included a structured and validated questionnaire made into a Google survey electronic form. The structured questionnaire for this educational research was prepared based on the principles and recommendations in UNESCO’s quantitative research methods in educational planning by the International Institute for Educational Planning [6]. Initial data were obtained from experts to formulate a questionnaire that was piloted with a representative cohort, then analyzed and improved using the feedback. The google form is a 58-field questionnaire with 10 sections including three qualitative questions and two consent sections, which took respondents an average of 7-12 minutes to complete.

After the introductory overview session, intended to help the respondents give voluntary informed consent by selecting a “yes” option, which led them to the rest of the questions, those who selected the “no” option were taken straight to the submit button. The rest of the sections were targeted as obtaining primary responses on participant’s background information, their reported exposure to the AT, their general perceptions of the AT and similar digital tools, their opinion of the utility of the AT as a teaching/learning tool, their experiential positions or opinion of factors influencing the acceptance of the Anatomage, their perception of the effectiveness of the Anatomage in meeting objectives of the three main learning domains (cognitive, affective, and psychomotor), and their understanding of the contexts or situations in which the Anatomage can be used as a medical education tool. The last three qualitative questions were a quest to get a deeper opinion of a larger pool of the study population on factors that do/might hinder or promote the acceptance of the AT in their current institution, as well as their individual opinion on the use of digital tools such as the AT in the training of medical students. The closing consent helped the team to obtain each participant's consent on being contacted to participate in either the focus group discussion and/or the in-depth interview as the case might be. A separate report captured the qualitative findings. 

Sample size determination

A total population sample of the target institution was reached for the quantitative survey such that all consenting individuals ultimately participate in the research. This was adopted in consideration of the estimated small population of anatomy teachers in the continent that met the inclusion criteria.

Data collection, management, and analysis

The quantitative data were collected using the online questionnaire in protected Google forms which was set not to allow more than one response from each participant. The quantitative data from the survey responses were downloaded in Excel format and prepared into a clean usable dataset using a detailed matching codebook prepared based on the variables and values from the questionnaire questions. The questionnaire had nominal, categorical, and continuous data variables but the only continuous variable was converted to categorical form for ease of analysis while the qualitative information was collected in text form. The whole data were subsequently stored in this format and uploaded onto SPSS version 23 (IBM Corp, Armonk, NY) for onward descriptive analysis using mainly frequency and cross-tab functions. The plan was that whenever necessary, and for inferential statistics, the p-value will be set at <0.05. The location data were mapped using QGIS version 3.14. Google sheets were also utilized to obtain bar charts, and other analytical presentation formats deemed best to present our findings.

Informed consent and confidentiality statement

The study survey questionnaire was prepared such that it gave participants double informed consent opportunities. The first consent opportunity gave participants access to the survey questions, and the second consent gave them the opportunity to register informed consent to be contacted by the research team for the subsequent qualitative sessions(s). The communications with the participants and recruitment of the participants were done with due recourse to ethical codes of respect for the dignity of participants, privacy and independence in voluntary choice, and sufficiently eliminating any form of power dynamics by authorities to influence subordinates. The Institutional Review Board of the University of Global Health Equity (UGHE) reviewed and approved the research (UGHE IRB #0085).

## Results

Recall that this study was targeted at getting an African version of the narratives, views, reported experiences, and perspectives of African anatomy teachers to sufficiently find answers to these three objectives:

1. To determine the perception of Anatomage technology by anatomy teachers in medical and allied health institutions in Africa and the influencing factors.

2. To determine the level of acceptance of Anatomage technology by medical school teachers.

3. To investigate factors that influence acceptance.

Our findings are presented thematically with each theme being a summary of the responses to questions on background, reported exposure to the AT, general perceptions of the AT and similar digital tools, their opinion of the utility of the AT as a teaching/learning tool, experiential positions or opinion of factors influencing the acceptance of the Anatomage, their perception of the effectiveness of the Anatomage in meeting objectives of the three main learning domains (cognitive, affective, and psychomotor), and their understanding of the contexts or situations in which the Anatomage can be used as a medical education tool. The findings from the last three open-ended questions on the respondent’s individual deeper opinion of factors that do/might hinder or promote the acceptance of the AT in their current institution as well as their individual opinion on the use of digital tools to teach anatomy is also summarized thematically.

Participants' background

**Figure 1:**
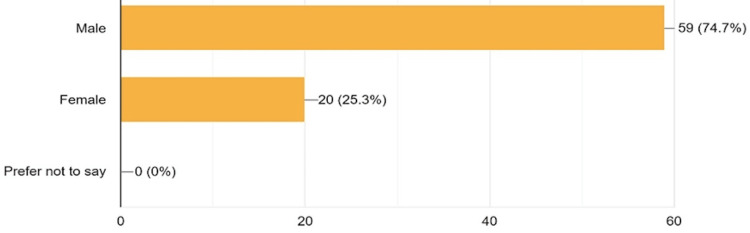
Gender Chart of Participants Approximately three-fourths of participants were males

A total of 79 consenting anatomy teachers who work primarily in 10 countries with a gender distribution proportion of 1:3 females to males (see Figure 1) and at 14 medical schools across Western, Eastern, Southern, Central, and Northern African regions participated in the online quantitative study. Approximately 25% of them are females. The response rate was ≈100%. Interestingly, 54.4% of these respondents are within the age range of 30-39 years, while 39.3% of them are aged 40-59 years. In the distribution of highest academic qualifications, PhDs or doctorate holders make up 43%, Master’s degree holders accounted for 46.8%, while 3.7% of them have a medical degree. Almost 90% of respondents have taught anatomy for less than 20 years (see Table 1). Ten representative African countries participated in the study (Table 2). 

**Table 1:** Approximate Number of Years Spent as Anatomy Teacher Categorized

Years Category	Frequency (n)	Percentage
0-9 years	42	53.2
10-19 years	29	36.7
20-29 years	5	6.3
30-39 years	2	2.5
>50 years	1	1.3
Total	79	100

**Table 2:** Breakdown of Participants by Country and Regions in Africa

S/N	Country of Residence	Participation		Region	Participation	
		Frequency (n)	Percentage		Frequency (n)	Percentage
1	Ghana	4	5	West Africa	20	25.3
2	Nigeria	16	20.3			
3	Ethiopia	18	22.8	East Africa	45	57
4	Kenya	9	11.4			
5	Rwanda	18	22.8			
6	Namibia	2	2.5	Southern Africa	8	10.1
7	South Africa	5	6.3			
8	Zambia	1	1.3			
9	Egypt	5	6.3	North Africa	5	6.3
10	Sudan	1	1.3	Central Africa	1	1.3
	Total	79	100		79	100

Represented countries and approximate years of teaching anatomy

**Figure 2:**
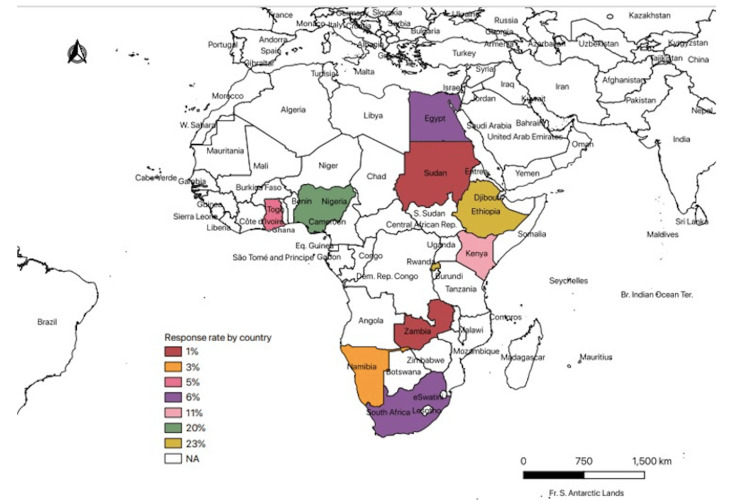
The Map of Africa Showing the Proportional Distribution of Respondents in Countries (Only Countries in Color Participated).

In terms of geographical location, the participants responded from Ghana (5.1%) and Nigeria (20.3%) in west Africa; Rwanda (22.8%), Ethiopia (22.8%), and Kenya (11.4%) from the East African region; Namibia (2.5%), Zambia (1.3%), and South Africa (6.3%) from the Southern African region; Sudan (1.3%) from Central Africa; and Egypt (6.3%) from Northern Africa (see Map in Figure 2). Most respondents had taught anatomy for less than 20 years.

A further breakdown of the respondents by their regional location in Africa shows a representation of East Africa (57%), West Africa (25.3%), Southern Africa (10.1%), North Africa (6.3%), and Central Africa (1.3%), respectively, in descending order of magnitude (see Figure 2 and Table 2).

Breakdown of participation in the Anatomage study by country and regions in Africa

On exposure to the AT, 54.4% of the participants reported that they have seen the tool whereas only 20.3% of the participants have used it as an educator or instructor to teach students. Interestingly, 96.2% of the participants have never used it as a learner (see Table 3).

**Table 3:** Participants’ Responses to Questions on Their Exposure to the Anatomage Table, Length of Use of the Anatomage Table, and Self-Rating of Mastery on Anatomage Use

Responses	Seen Anatomage Table		Used Anatomage as a Learner		Used Anatomage as an Educator/Instructor	
	Frequency (n)	Percentage	Frequency (n)	Percentage	Frequency (n)	Percentage
No	35	44.3	76	96.2	61	77.2
Yes	43	54.4	3	3.8	16	20.3
Not sure	1	1.3	0	0	2	2.5
Total	79	100	79	100	79	100

**Figure 3:**
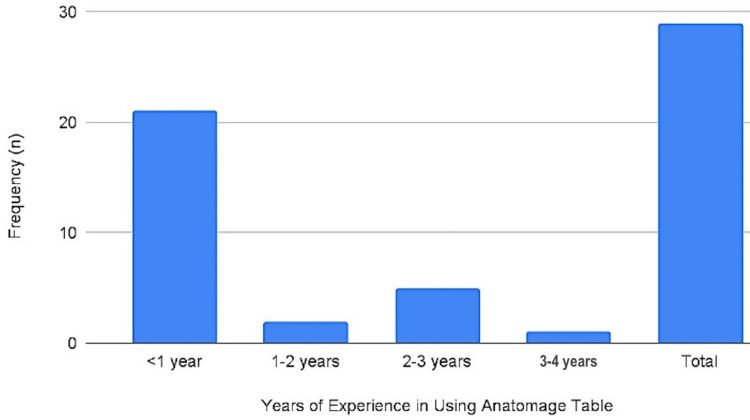
A Chart of Participants’ Years of Experience in Using the Anatomage.

When participants were asked how long they have been using the Anatomage, a total of 29 reported being users. Twenty-one of them have been using it for less than one year, two have used it for at least one year but not more than two years, five have been active users for 2-3 years, whereas only one participant reported being an active user for more than three years but not more than four years (see Figure 3). A total of 23 (29.1%) respondents reported that they have some level of mastery in using the AT when asked to grade their mastery of it. 6.3% of all the participants reported having a high mastery of this tool, whereas 12% and 6% reported that they have an average mastery and low mastery, respectively (Table 4).

**Table 4:** Participants’ Self-Rating of Their Anatomage Usage Mastery

Responses	Frequency (n)	Percentage
Low	6	7.6
Average	12	15.2
High	5	6.3
Total	23	29.1

Interest in knowing more about the AT

The participant's responses to the four questions on their interest in knowing more about the AT shows that 77.2% reported reading about the Anatomage at some point, 69.6% have watched at least a video on Anatomage use, 91.1% reported that they are enthusiastic about learning more about the tool, and 83.5% of respondents look forward enthusiastically to using the tool in the future (see Table 5). However, only 17.7% of the participants reported receiving any form of formal training on Anatomage use.

**Table 5:** Participants' Show of Interest in Knowing More About the Anatomage Table

Responses	Read About Anatomage Use		Watched At Least 1 Video on Anatomage Use		Enthusiastic to Learn More About Anatomage as an Educator		Enthusiastic About Using Anatomage in Future		Received Formal Training on Anatomage Use	
	Frequency (n)	%	Frequency (n)	%	Frequency (n)	%	Frequency (n)	%	Frequency (n)	%
No	15	19	22	27.8	2	2.5	2	2.5	64	81
Yes	61	77.2	55	69.6	72	91.1	66	83.5	14	17.7
Not sure	3	3.8	2	2.5	5	6.3	11	14	1	1.3
Total	79	100	79	100	79	100	79	100	79	100

Institutional ownership, plan of procurement, utilization of the AT, and support for digital tools

**Figure 4:**
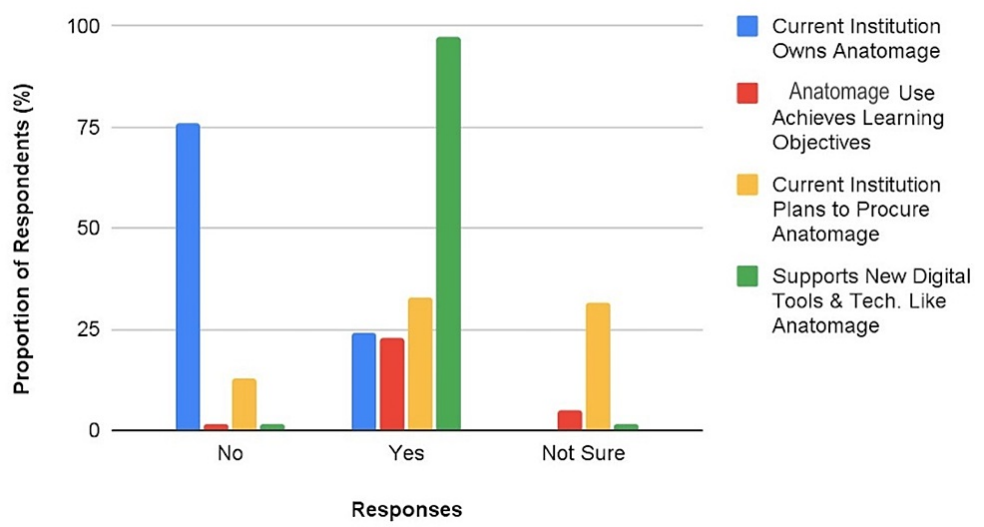
A Chart on Reported Institutional Ownership, Plan of Procurement, and Utilization of the Anatomage Table Cum Support for Digital Tools Like Anatomage

Across the surveyed population, 19 participants (24.1%) reported that their current medical school has the AT and 18 of them think that the Anatomage use is helping their department to achieve learning objectives. On the users’ level of satisfaction with the use of the AT at their institution, 44.4% and 40.7% of them reported being satisfied and very satisfied, respectively. On the other hand, 32.9% of the participants admitted that their current institution has a plan of procuring AT. Interestingly, individual support for ideas about introducing innovative tools and technologies including Anatomage into anatomy teaching was reported by 97.5% of the respondents (see chart in Figure 4).

Participants’ rating of the general usefulness of the AT

**Figure 5:**
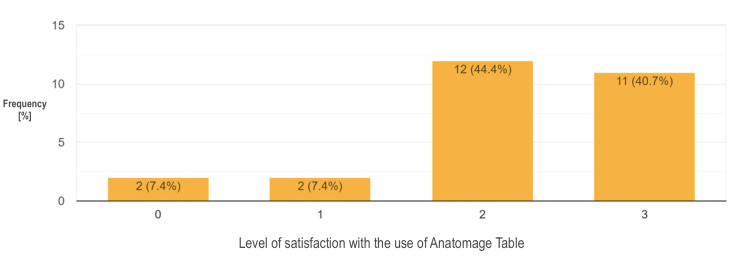
A Chart of Users’ Level of Satisfaction With the Use of the Anatomage Table at Their Institution (Where: 0 = Not Satisfied, 1 = Fairly Satisfied, 2 = Satisfied, 3 = Very Satisfied).

The participants' rating of their level of agreement with the statement that the AT is a useful tool for teaching medical and allied health students showed that 54.4% of them strongly agreed while 27.8% just agreed. On its consideration as an innovative digital anatomy teaching aid, 64.6% of the respondents were strongly in agreement with it while 24% simply agreed that it is. Also, 48.1% strongly agreed while 38% simply agreed that AT technology innovation can significantly contribute to the advancement of medical education. However, 21.5% of the participants were neutral in their stand on whether the Anatomage can present accurate information about the human body anatomy, and those who agreed strongly or just agreed with it accounted for 34.2% and 36.7%, respectively (see Table 6). Also, most anatomists were generally satisfied with the use of the AT (Figure 5).

**Table 6:** Rated Perception of the Usefulness of the Anatomage Table

Rating	Useful for Teaching Anatomy to Medical and Allied Health Students		An Innovative Digital Anatomy Teaching Aid		Can Present Accurate Information About the Human Body Anatomy		Can Contribute Significantly to the Advancement of Medical Education	
	Frequency (n)	%	Frequency (n)	%	Frequency (n)	%	Frequency (n)	%
Strongly disagree	2	2.5	1	1.3	1	1.3	1	1.3
Disagree	4	5.1	1	1.3	5	6.3	2	2.5
Neutral	8	10.1	7	8.9	17	21.5	8	10.1
Agree	22	27.8	19	24	29	36.7	30	38
Strongly agree	43	54.4	51	64.6	27	34.2	38	48.1
Total	79	100	79	100	79	100	79	100

Participants’ expression of their level of agreement with the effectiveness of the AT in specific uses and applications in teaching anatomy

**Figure 6:**
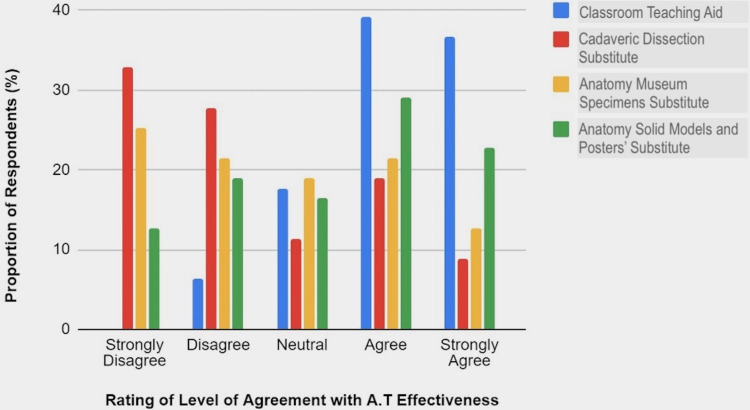
Chart Comparing Participants’ Levels of Agreement With Stated Specific Usefulness or Applications of the Anatomage Table in the Teaching of Anatomy.

The participants also expressed their level of agreement with statements on the effectiveness of the AT in specific areas of its usefulness or application. On the effectiveness of AT as a classroom teaching aid, 17.7% were neutral whereas those who simply agreed or strongly disagreed accounted for 39.2% and 36.7%, respectively. When asked if AT can fully substitute for cadaveric dissection in the anatomy laboratory, 32.9% and 27.8% of the respondents strongly disagreed and simply disagreed, respectively, and 19% of them simply agreed. The pattern of response to the question if AT can fully replace anatomy museum specimens such as pots and plastinates was such that 12.7% of the participants strongly agreed, 21.5% each simply in agreement or disagreement, and 25.3% of them strongly disagreed with it. While 19% of the respondents disagreed with the effectiveness of AT to substitute for anatomy models and posters, 29.1% and 22.8% of them were simply in agreement and strongly agreed, respectively (see Figure 6).

Participants’ expression of their level of agreement with the effectiveness of using the AT to teach anatomy subjects

**Figure 7:**
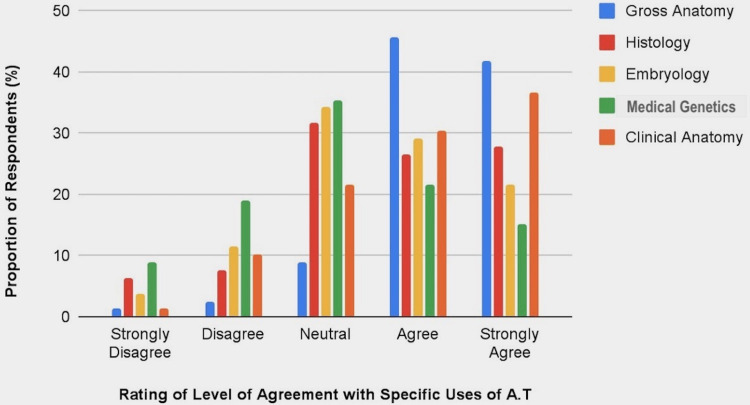
Chart of Participants’ Rating of Their Level of Agreement With Effectiveness of Using Anatomage to Teach Various Anatomy Subjects. A.T,  Anatomage table.

The variations in the participants' level of agreement with how effective the Anatomage is or could be when used to teach various anatomy subjects was also observed. Most of them took a neutral stand, especially on histology (31.6%), embryology (34.2%), and medical genetics (35.4%). On the effectiveness of using Anatomage to teach gross anatomy, 45.6% of the respondents were simply in agreement while 41.8% of them strongly agreed. Though 21.5% of the respondents were neutral on how effective Anatomage could be in teaching clinical anatomy subjects such as radiology and surgical skills, 30.4% and 36.7% of them were in agreement and in strong agreement, respectively. On histology and embryology, 27.8% and 21.5% were in strong agreement, whereas 26.6% and 29.1% were simply in agreement, respectively (see Figure 7).

Perceived effectiveness of Anatomage in achieving the objectives in the three domains of learning

**Figure 8:**
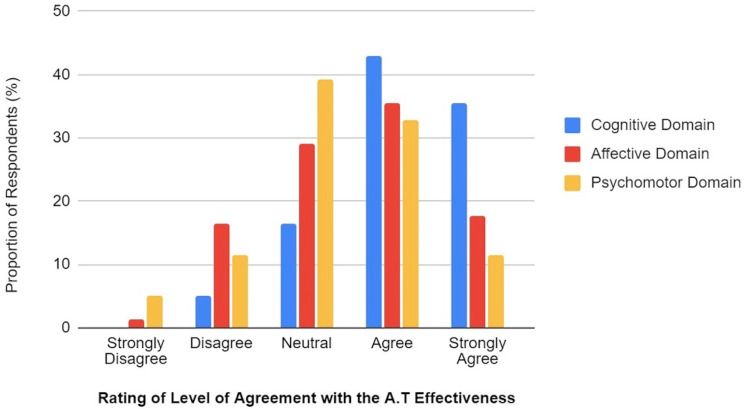
The Participants’ Expressed Perception of Their Level of Agreement With the Effectiveness of Anatomage Table in Achieving the Cognitive, Affective, and Psychomotor Learning Objective Domains.

They were also asked to rate their level of agreement with the Anatomage being effective and helpful at achieving the objectives of learning in the cognitive (knowledge or mental skills), the affective (emotional or attitude), and the psychomotor (physical skills) domains. Whereas 16.5% took a neutral stand on its effectiveness in the cognitive domain, 29.1% and 39.2% were neutral about its usefulness in realizing the affective and the psychomotor learning objectives, respectively. However, the pattern of response to the question of its efficiency in achieving learning objectives in the affective and psychomotor domains was such that 35.4% and 32.9%, respectively, simply agreed, while 17.7% and 11.4%, respectively, of the respondents were strongly in agreement (see Figure 8).

Factors that might support acceptance of the AT technology compared to cadaver-based learning

**Figure 9:**
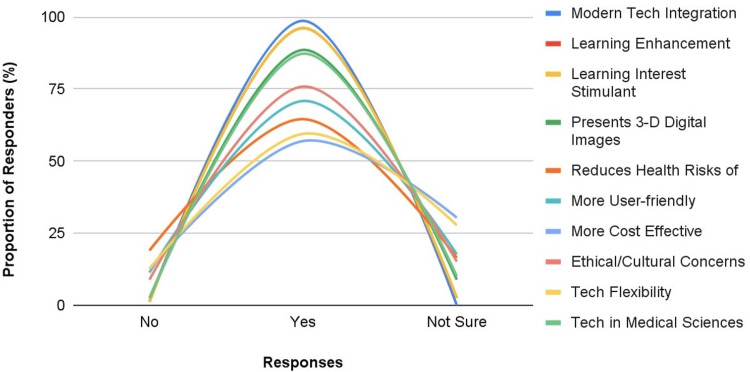
Participants’ Perception of the Factors That Might Support Acceptance of the Anatomage Table Technology Over Cadaver-Based Learning.

A number of factors identified from the literature as being in support of the acceptance of the AT over cadaver-based learning got the result of responses from the study participants across Africa (see Figure 9). The proportion of respondents who agreed to a need to use Anatomage as a modern technology for teaching based on its potential to enhance learning and stimulate interest in learning were 98.7%, 96.2%, and 96.2%, respectively. Some saw the Anatomage as a means of embracing the global culture of technology-in-medical sciences (87.3%).

A varied percentage of our respondents agreed that the Anatomage has the potentials of presenting anatomy in 3D life-like images in digital form (88.6%), reducing exposure to the contagious health risks of cadavers (64.6%), and being more cost-effective in the long run compared to the cost of processing and preserving cadavers (57%). Many of the participants think that there are no ethical and cultural concerns associated with Anatomage use when compared to cadavers (75.9%). A smaller proportion agreed that the Anatomage is more technologically flexible compared to the stereotypical cadaveric dissection (59.5%), and that students would find it more user-friendly than embalmed cadavers (70.9%).

Participants’ perception of the contexts of Anatomage usage for teaching anatomy - a suitable substitute or a complementary tool to cadaver

**Figure 10:**
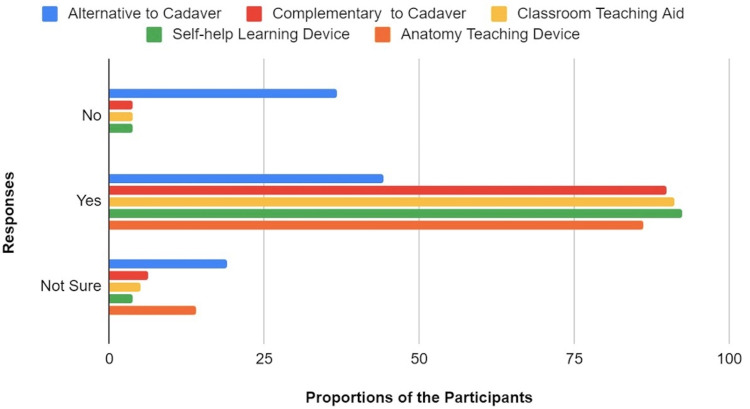
A Chart of Participants' Perception of the Contexts of Anatomage Usage for Teaching Anatomy.

One of the most important outcomes of this study is to understand what the take of the respondents is, on the question of whether the Anatomage is a suitable alternative to cadaveric dissection or should just be a complementary method to cadaveric dissection (see Figure 10). Those who think that Anatomage can suitably replace cadaver-based learning were 44.3% of the participants, whereas 89.9% of the respondents rather think that it should be a complementary anatomy teaching tool to the cadaver. There was a reduced variation in the proportion of the participants that affirmed the Anatomage is or could be a good classroom teaching aid (91.1%), a student self-help learning device (92.4%), and a great device for teaching anatomy content for any related field of medical science (86.1%). There is a consensus that Anatomage is a useful EdTech for anatomists, educators, and learners; however, respondents would not largely agree that it should absolutely replace cadaveric dissections (Figure 10).

## Discussion

Demography and professional attributes of anatomy educators

Most respondents were males. In terms of professional cadres, participants were almost equally divided between low/middle and senior career-level academics. The distribution of participants who were representative anatomists and medical educators in African institutions is of importance for specific reasons. First, it speaks to the generation, that is anatomists and medical educators belong by age and duration for which they have practiced the science of anatomy. It also speaks to the training background that they had and how these could have influenced their impressions about EdTech and the use of technology and innovations generally in education.

It is a good thing that the proportion of African anatomists is almost equally divided between the older generation of medical educators and the younger generation, as this would provide a good mix that could enrich perspectives and provide a balance between such perspectives. It is, however, important to note that, typically, the older generations are usually in positions of academic leadership, hence making critical decisions about institutional infrastructure setup, curricular and program philosophies, and mainstream pedagogies, which altogether would influence the culture of learning and teaching in their institutions. Bearing these realities in mind, therefore, it is important to continually organize programs that educate and enlighten anatomists and medical educators in Africa on current developments, advancements, and trends toward ensuring that stakeholders are in tune with global developments. Such enlightenment will help to align with developments and best practices. On the other hand, having a good proportion of agile, tech-enthusiastic, and tech-inclined-and complaint anatomists and medical educators will continuously help to promote a tech culture and the need to embrace advancement, especially in terms of EdTech pedagogical advancements. We would, therefore, consider that anatomical science and medical education on the African continent has quality prospects because of the quality mix of the anatomists and educators in terms of their demographical divisions into early and mid-career professionals and senior-level career professionals. We think that this factor will help to maintain a balance. 

Duration of exposure to the EdTech ranged between one and four years. Most respondents would only rate their exposure and competencies with the use of this tool as average. It is also important to observe that most respondents are just getting exposed to educational technologies, especially AT. Exposure altogether ranged between one and four years. While one could wish that Africa altogether had earlier embraced EdTech, it is a good sign that the tech culture is currently being cultivated on the African continent among anatomists and medical educators. Also, the inferences from their responses would show that this trend will continue to grow, and expectedly rapidly, we consider this to be another plus for the anatomists on the African continent. A tech-inclined mindset, as against apathy and aversion to technology, will definitely help the continent to leapfrog in terms of development, especially in the area of anatomical sciences and medical education, and by extension in terms of the qualities of the delivery of care in the continent. It is important to emphasize the fact that technology has become an integral tool toward achieving quality advancements in various walks of life. In the same vein, educational technologies or EdTech have become integral to advancements in education, in Africa and globally [7-12].

Only one out of five anatomists or medical educators currently use the Anatomage and only about 3% used it during their training as learners. These statistics will show that most current practicing anatomists and medical educators did not use the AT while they were training as anatomists, medics, or medical educators. This is not unexpected as this technology is relatively new and has come as a product of recent advancements in educational technology. Currently, only 20% of medical educators could ascertain that they could use the technology. While this remains a current reality, it is a pointer to the need to provide training opportunities for anatomists and medical educators on the continent to enable them to acquire skills and competencies to use this technology and other available forms of EdTechs. Such efforts can include conferences on medical education and EdTechs as well as training the trainer workshops. It might be important to establish medical education hubs that might serve as centers of excellence, in countries and regions where medical educators can be trained in the various aspects of medical education and with adequate emphasis on the use of educational technologies of various types. This would further the call for the need for quality collaborations and cooperation among stakeholders in medical education as well as tertiary education on the African continent.

Information about teachers' perceptions and the influencing factors

Anatomists are positive about acquiring and using EdTech and innovations. Most anatomists and medical educators agree/strongly agree that AT as an EdTech offers significant values and benefits when used to support medical education. It is quite encouraging that African anatomists and medical educators are positive about not just acquiring competencies and skills to use educational technologies but also acquiring the technologies and facilities to support their teaching and training activities. This will further imply that African anatomists and medical educators have come to appreciate the role that educational technologies and innovations can play in training students and professionals; this is also in line with global trends and current practices. For example, the COVID-19 event between the years 2020 and 2021 provided avenues for several institutions globally to explore opportunities to sustain education using various types of technologies and innovations. Thankfully, there are several reports of successes in institutions whereby stakeholders in medical education had opted for various technologies to support the delivery of medical education with optimal levels of performance being reported and recorded. Part of the benefits of enshrining a tech culture in the training of medical and allied health professionals would also include that we would be producing tech-compliant medical and health professionals. African anatomists and medical educators are in alignment with global trends on the embrace of a tech culture This is also in line with global trends and informed opinions across the world [13-17].

Information about the teachers’ acceptance of Anatomage Table

Acceptance is broadly positive; the consensus is to use AT as a teaching aid and a complementary EdTech to support anatomy teaching and medical education. The majority are averred to using the EdTech to totally replace actual cadaveric dissection. In terms of the sub-divisions or branches of anatomical sciences, the use of Anatomage favors the contexts of gross anatomy and clinical anatomy. Most respondents believed cadaver dissection should be sustained as it remained, so to say, a traditional pedagogical practice that is almost synonymous with a practice of anatomy or anatomical science. This is understandable as a good proportion of participants were also trained using cadaveric dissections as an anatomical practice. It is equally important to state that the practice of cadaver dissection is as old as the practice of anatomy itself; hence, it has remained tested and trusted over time, and in many climes across the world anatomists have continuously promoted the need to sustain cadaveric dissection or its other variants such as prosections. The position of African anatomists and medical educators who participated in the current study, therefore, remained that cadaver dissection should be sustained whereas the use of Anatomage should be complementary as it could add significant value to the teaching of anatomical sciences to medical and allied health sciences students.

Most respondents largely believed that AT use would enhance learning in the cognitive, affective, and psychomotor domains, with the weights of support in this same order. It is important to note that since the cognitive domain emphasizes the acquisition of knowledge and understanding of concepts and phenomena, a high-quality presentation of the human anatomy will enhance the cognitive understanding of concepts and phenomena about human morphology as such. This would most likely explain why most stakeholders would think that the most significant benefit of the use of EdTech and more specifically the AT would be in the cognitive domain. Embracing a culture of EdTech or being technologically compliant is one affective benefit that the use of the AT could offer trainees, and rightly so. While most would generally still agree that this EdTech has benefits in the psychomotor domain of learning, they believe that it is the least ranked out of the three primary domains of learning that could benefit from EdTech. It is, however, important to note that the perspective from which stakeholders view the use of this technology matters in this regard. While it is true that merely using the AT to present 3D representations of the human body will most likely enhance learning most significantly in the cognitive domain, it is important to note that it is possible to practically use this technology to simulate clinical procedures and physiological phenomena, superimposed or placed within the context of the morphological architecture of the body, which can also offer a quality psychomotor advantage, hence helping learners to acquire specific skills or to enhance their learning in the skill or psychomotor domain as well. This would further point out the fact that the level of competency of the trainer who uses the AT, and by extension educational technologies to deliver medical education and training to students would matter as to what is being emphasized and what is achievable with the use of the technology. Once again, one might advocate for training the trainers programs that emphasize EdTech on the African continent and integrate and standardize the use of EdTech [18].

Information about factors that influence acceptance or otherwise

Promotion of a tech culture through EdTech integration, stimulating learners’ interest, and presenting images in 3D formats are the most important factors that promoted educators’ interest in the AT, the least factor being that it might be cost-effective. Most educators who participated would think that it is a great teaching aid, a complementary learning tool that might also serve as a classroom facility as well as a self-help facility for learners. It is encouraging to realize that stakeholders in the African educational industry, especially the anatomists and medical educators, are tech-inclined [19,20]. It is equally positive that these stakeholders found reasons and a good purpose to embrace EdTech. It is, therefore, important to integrate this newfound ideology, so to say, into the mainstream of medical education delivery and, by extension, practice. The fact that the cost implication might be a prohibitive factor needs to be well addressed.

While respondents did not necessarily think that their institutions could not afford the technology, they simply would think that the institutions would not prioritize such an investment. There is, therefore, a need to engage stakeholders, especially the leadership and decision makers in the educational industry, to place adequate value on educational technology and innovation and as such be willing to invest adequately in the procurement and use of educational technologies and innovation to support the training of scientists and medical professionals. It will also be very helpful if the producers of this technology will put in place programs that can promote a tech culture on the African continent, and to provide engagement avenues and opportunities whereby they might have to engage stakeholders and leadership on how best to help their institutions. Such efforts can possibly include supporting them to acquire and use EdTechs. We equally advocate for a need to close the relationship and communication gap between the EdTech-producing industry and the education industry. There might be a need for increasing collaborations between the EdTech industry, medical schools, and tertiary education institutions in general. It would not be out of place for academics and researchers to be involved in the design and development of EdTech, and for stakeholders in the educational tech industry to also venture into academic endeavors. Quality collaboration will facilitate quality conversations and relationships that would be mutually beneficial to both industries [21-24].

Study's limitations

The study is a cross-sectional study, hence it could not have provided extensive information on trends and advancements relative to the past. It will require a longitudinal study in the future to study trends and advancements over time. Furthermore, this study was conducted virtually due to limited funding and COVID-19-related travel restrictions. The restrictions limited physical contacts with people in the representative countries; also, limited funding favored the use of electronic questionnaires that could be administered to the target audience on the continent without physically traveling to the institutions. 

## Conclusions

African anatomists and medical educators are receptive and enthusiastic about the use and integration of Anatomage as an EdTech to support the teaching of anatomy and related basic medical sciences to students and trainees. They would think that the benefits of the EdTech include its alignment with advancements in medical and health education, and the need to enhance the delivery of care. Therefore, there is a need for the promotion of EdTech and educational innovations, and to adopt t tech culture. They also agreed that it should be complementary EdTech to enhance teaching, training, and learning and not replace cadaveric dissection. Their responses also suggested that they think the cost factor might limit their abilities to acquire this technology. Tech competence and exposure might at best be rated average, and this would warrant the need to promote EdTech and train users and potential users. This might be an indication that Africa is receptive to EdTech and innovations for medical education. 
